# Self-efficacy in Thought Control , not Positive Gains, Mediates Effect of Benefit-Finding Intervention for Carers

**DOI:** 10.1093/geroni/igab046.2970

**Published:** 2021-12-17

**Authors:** Sheung-Tak Cheng

**Affiliations:** Education University of Hong Kong, The Education University of Hong Kong, Hong Kong

## Abstract

This study examines the therapeutic mechanism of the benefit-finding therapeutic (BFT) intervention that used cognitive reappraisal and alternative thinking to construct positive aspects of caregiving (PAC), in a cluster-randomized controlled trial for Alzheimer caregivers. 42 caregivers received BFT whereas 87 received psychoeducation as control. Both interventions were held in groups. Depressive symptoms and global burden were outcomes measured at baseline, postintervention, and 4- and 10-month follow-up. Mediators considered included PAC and three self-efficacies—controlling upsetting thoughts (SE-CUT), responding to disruptive behaviors, and obtaining respite. Using mixed-effects regression, we demonstrated that benefit-finding increased caregivers’ PAC and SE-CUT, but that only SE-CUT uniquely predicted depressive symptoms and global burden longitudinally. Path analyses with bootstrapped confidence intervals showed that SE-CUT change from baseline to postintervention mediated intervention effects on depressive symptoms, but not global burden, at both follow-ups. No mediation effects were found for PAC and the other self-efficacies. As a conclusion, The BFT effect on depressive symptoms was partly accounted for by improvement in SE-CUT. The therapeutic mechanism for the effect on burden remained unknown. The study sheds light on the importance of actively promoting positive caregiver functioning.

